# Knowledge, Attitudes, and Practices Regarding Respiratory Syncytial Virus Vaccine for Older Adults Among Family Medicine Practitioners at King Khalid University Hospital, Riyadh, Saudi Arabia

**DOI:** 10.7759/cureus.101668

**Published:** 2026-01-16

**Authors:** Abdullah M Alzahrani, Sulaiman A Alshammari, Norah Y Alawlah, Sarah A Alhamlan, Abdulrahman H Alduhayyim, Majed A Almasaoud, Sulaiman F Alzomia, Rakan M Alghonaim, Maha A Aljohani

**Affiliations:** 1 Department of Family and Community Medicine, King Saud University Medical City, Riyadh, SAU; 2 College of Medicine, King Saud University, Riyadh, SAU

**Keywords:** barriers, family medicine, knowledge, older adults, respiratory syncytial virus, respiratory syncytial virus vaccine

## Abstract

Objectives: The introduction of respiratory syncytial virus (RSV) vaccines for older adults has created new implementation demands in primary care. This study aimed to assess family medicine practitioners' knowledge, attitudes, and self-reported practices regarding RSV vaccination in older adults and to identify the factors associated with these outcomes.

Methods: A cross-sectional analytical study was conducted with 120 family medicine practitioners. They completed a validated structured questionnaire regarding their background, knowledge, attitudes, practices, and perceived barriers to RSV vaccination. Descriptive statistics, correlation analyses, and chi-squared tests were used to examine the links between practitioner characteristics and knowledge, attitudes, and practices.

Results: Attitudes were somewhat positive (35.8%), but many were not confident discussing RSV vaccination. Actual practices were limited (29.2%); only 14.2% prescribed or administered RSV vaccines, and 49.2% had never discussed vaccination. The primary barrier was a lack of provider awareness (80.8%). Knowledge and attitude scores were closely linked, and better practices were associated with greater knowledge, more positive attitudes, job position, and longer experience. Family physicians had favorable knowledge and attitudes regarding RSV vaccination.

Conclusions: We found a misalignment between awareness, confidence, and self-reported clinical practice regarding RSV vaccination in primary care. Implementation barriers appear to extend beyond vaccine availability and include gaps in clinician preparedness and role clarity, highlighting the need for targeted implementation supports, such as clear clinical responsibility, decision workflows, and patient communication tools, alongside education.

## Introduction

Respiratory syncytial virus (RSV) is a well-established cause of respiratory infection in children and an increasingly recognized cause of severe respiratory disease in older adults, leading to severe outcomes, such as hospitalization and mortality [1,2]. Older adults, particularly those with comorbid conditions, such as cardiovascular and pulmonary diseases, are at an increased risk of severe RSV infection [3-5], and studies have shown that RSV-related hospitalization and mortality rates in older adults are comparable to those of influenza [2,6]. Despite this burden, RSV remains under-recognized as a clinically significant pathogen in older adults [7].

RSV vaccine development has accelerated recently, with several vaccines in the advanced stages of clinical trials and others already approved for use in older adults [8]. The availability of RSV vaccines presents an opportunity to reduce disease burden among older adults, particularly in settings such as Saudi Arabia, where surveillance data on RSV in older populations remain limited [9]. Therefore, adapting vaccination programs to address the specific needs of these vulnerable groups is crucial for enhancing preventive efforts and reducing RSV-related morbidity and mortality [10]. Primary care physicians (PCPs) are essential to ensure the success of such vaccination programs [11].

PCPs' knowledge and clinical perceptions regarding RSV and its prevention are key determinants of vaccine recommendation and uptake in adult populations. A study conducted between February and March 2017, which surveyed 930 PCPs across national networks, found that a significant number lacked sufficient knowledge regarding RSV in adults. Among respondents who reported caring for RSV patients (n=317), 73% and 57% reported that, in patients ≥ 50 years, influenza is generally more severe than RSV and that they rarely considered RSV as a potential pathogen, respectively. Most (61%) agreed that they do not test for RSV because there is no treatment. The most commonly reported anticipated barriers to RSV vaccination were potential out-of-pocket expenses for patients if the vaccine was not covered by insurance (93%) and lack of reimbursement for vaccination (74%) [12]. This knowledge gap can hinder the proactive recommendation of RSV vaccines to older patients, which is a key driver for vaccine uptake.

Vaccine hesitance has increased in many countries. The World Health Organization (WHO) defines it as suboptimal vaccination coverage resulting from delays in vaccine acceptance or refusal despite the availability of vaccination services [13]. A Canadian study demonstrated that the most frequently reported barriers were lack of vaccine information (41%), lack of access to vaccination (38%), fear of adverse reactions (38%), financial reasons (29%), lack of awareness of vaccine existence (29%), antivaccine sentiments (24%), the notion that older adults do not need vaccination (18%), misconceptions on vaccine effectiveness (12%), potential sexual health promotion stigma (6%), and fear of needles (3%) [14]. Additionally, one of the challenges faced by family physicians is the limited opportunity for them to discuss and recommend vaccines, primarily because of the short duration of consultations, which often last for approximately 10 minutes. As patients usually visit their general practitioner (GP) for urgent health concerns, it can be difficult to prioritize discussions about adult vaccination over more pressing medical issues [15]. In Saudi Arabia, there is limited research on PCP attitudes toward RSV vaccination; however, studies on influenza and pneumococcal vaccines suggest that physician education and clear guidelines can enhance vaccine recommendation practices [16,17].

In Saudi Arabia, vaccination practices for older adults primarily focus on influenza and pneumococcal vaccines [16,17]. A similar pattern is anticipated for RSV vaccination, with PCPs expected to play a pivotal role in identifying eligible individuals and promoting vaccine uptake. According to recent Centers for Disease Control and Prevention (CDC) recommendations, a single RSV vaccine dose reduces the risk of severe RSV infection and hospitalization among adults aged ≥75 years and 50-74 years who are at increased risk for severe disease [18].

In Saudi Arabia, where PCPs play a central role in preventive care delivery, understanding their knowledge, attitudes, and practices related to RSV vaccination is essential to guiding national implementation efforts. Accordingly, this study aimed to evaluate family medicine practitioners' knowledge, attitudes, and practices regarding RSV vaccination in older adults and examine the demographic and professional factors associated with these outcomes.

## Materials and methods

Study design and setting

This quantitative, analytical, cross-sectional study was conducted at King Khalid University Hospital (KKUH) in Riyadh, Saudi Arabia. Data collection took place over four months from August to December 2025. Surveys were distributed electronically through online platforms.

Study population and sampling

The study population comprised all family medicine practitioners at KKUH, including residents, registrars, and consultants. Given the finite, accessible population size, a total population-sampling approach was employed. No formal sample size was calculated as the study aimed to capture responses from the entire available population. Approximately 150 eligible practitioners were invited; 120 completed responses were analyzed, yielding an estimated response rate of 80%.

Recruitment and data collection 

Participants were recruited through an online Google Forms survey that required approximately 2-3 minutes to complete. The survey began with an introductory section outlining the study's objectives and confidentiality assurances, followed by demographic items and questions assessing knowledge, attitudes, practices, and barriers related to RSV vaccination. Participation was voluntary, and informed consent was obtained upon agreement to complete the questionnaire. Responses were anonymous, with no identifying information such as names, contact details, or IP addresses collected. Once the data collection period ended, the survey link was disabled, and the data were securely stored for analysis. 

Inclusion and exclusion criteria 

Inclusion criteria comprised all family medicine practitioners at KKUH. Exclusion criteria applied to participants with technical difficulties or incomplete responses that prevented analysis.

Instrument development and validation 

A specific questionnaire was developed based on CDC international clinical guidance and recommendations for adult RSV vaccination [18] and WHO resources related to vaccine hesitancy [13]. Item generation was guided by the study objectives to assess family medicine practitioners' knowledge and awareness regarding the benefits and recommendations of RSV vaccination for older adults and to identify common attitudes, practices, and perceived barriers to RSV vaccine administration. Content validity was ensured through expert reviews by five specialists (three family physicians, one preventive medicine physician, and one infectious disease specialist), and items were refined for clarity and relevance based on their feedback. Pilot testing was conducted with 20 physicians to assess clarity and feasibility, and minor wording modifications were made accordingly. Internal consistency reliability was evaluated using Cronbach's alpha. The full study questionnaire is provided in the Appendices.

Ethical considerations 

Ethical approval was obtained from the King Saud University Medical City Institutional Review Board (IRB) (approval number: E-25-10046) prior to study initiation. Informed consent was obtained, and participants were assured of their right to withdraw from the study at any time without obligation. Confidentiality was ensured through anonymous data collection, with no identifying variables recorded, and no incentives or rewards were provided.

Questionnaire criteria

Participants' knowledge of the RSV vaccine was assessed using a seven-item questionnaire, with "true" coded as 1 and "false/I don't know" coded as 0 as the answer options. Question #6 is a negative question, where "false" is the correct answer (coded as 1). Total knowledge was calculated by adding all seven items. Scores ranging from 0 to 7 points were generated. Higher scores indicate greater knowledge about the RSV vaccine. Using 60% and 80% as cutoff points to categorize knowledge level for descriptive interpretation, participants were classified as having poor knowledge if their score was 0-3 (<60%), moderate knowledge if their score was 4-5 (60-80%), and good knowledge if their score was 6-7 (>80%) [19]. The reliability test for the knowledge questionnaire had a Cronbach's alpha of 0.751, indicating good internal consistency.

Participants' attitudes toward the RSV vaccine were assessed using a five-item questionnaire with 5-point Likert scale categories ranging from "strongly disagree" (coded as 1) to "strongly agree" (coded as 5). The total attitude score was calculated by adding all five items. Scores ranging from 5 to 25 points were obtained. Higher scores indicate more positive attitudes toward the RSV vaccine. Using 60% and 80% as cutoff points to categorize attitude level, participants were considered to have a negative attitude if their score was below 60% of the maximum score; further, 60-80% was deemed neutral, and above 80% was deemed positive [19]. A reliability test of the attitude questionnaire showed a Cronbach's alpha of 0.904, indicating excellent internal consistency.

For practice, given the early stage of RSV vaccine implementation, we used the question "Have you ever discussed RSV vaccination with an older patient?" as a proxy indicator of clinical engagement, with "yes" considered a favorable practice and "no" considered an unfavorable practice.

Statistical analysis

To ensure sufficient cell sizes for analysis, selected categorical variables were collapsed based on conceptual similarity, given skewed distributions and the relatively small sample size. Merging categories enables better comparisons between variables.

Categorical variables were described as counts and proportions (%), while continuous variables were presented as means and standard deviations. The relationships between demographic characteristics and knowledge, attitudes, and practices regarding the RSV vaccine among older adults were examined using Fisher's exact test. Differences in knowledge and attitude scores across the demographic characteristics of family medicine practitioners were analyzed using the Mann-Whitney U-test. Spearman's correlation was also used to assess the correlation between knowledge and attitudes. Normality tests were performed using the Shapiro-Wilk test. Based on Shapiro-Wilk testing and visual inspection of distribution plots, knowledge and attitude scores demonstrated non-normal distributions. Therefore, nonparametric tests were applied. Statistical significance was set at p<0.05. All statistical data were analyzed using IBM SPSS Statistics for Windows, Version 26.0 (IBM Corp., Armonk, New York, United States).

## Results

A total of 120 family medicine practitioners participated, with comparable representation of men (51.7%) and women (48.3%). Approximately 46% were under 30 years of age. Consultants constituted the largest group (41.7%), followed by residents (39.2%); only 3.3% were GPs. Half of the participants had less than five years of experience in family medicine (Table 1).

**Table 1:** Sociodemographic characteristics of the family medicine practitioners (n=120)

Study variables	N (%)
Age group	
<30 years	55 (45.8%)
30-35 years	18 (15%)
36-40 years	12 (10%)
41-45 years	17 (14.2%)
46-50 years	6 (5%)
>50 years	12 (10%)
Gender	
Male	62 (51.7%)
Female	58 (48.3%)
Job position	
Resident	47 (39.2%)
General practitioner	4 (3.3%)
Registrar	19 (15.8%)
Consultant	50 (41.7%)
Years of experience in family medicine	
<5 years	61 (50.8%)
5-10 years	22 (18.3%)
11-15 years	19 (15.8%)
16-20 years	4 (3.3%)
>20 years	14 (11.7%)

Three-quarters (75%) knew that RSV is a seasonal virus, and 87.5% understood that it poses risks to older adults. Nearly two-thirds (64.2%) were aware of the Ministry of Health's recommendations, whereas 57.5% understood that RSV vaccines could be administered along with influenza and COVID-19 vaccines. However, only 14.2% of participants correctly identified the number of approved vaccines. The mean knowledge score was 4.68±1.91 (out of 7), with 41.7% classified in the highest knowledge category based on predefined thresholds.

The total mean attitude score was 18.7 out of 25 points. Attitude levels were classified as positive in 35.8% of practicing physicians, neutral in 37.5%, and negative in 26.7%. Ratings were higher for statements indicating that RSV is a serious disease (mean=4.14) and that vaccination should be part of routine care (mean=4.04). In contrast, confidence in discussing RSV vaccination with patients was lower (mean=3.28).

Reported clinical practices were infrequent: 29.2% had discussed RSV vaccination with older patients, and only 14.2% had prescribed or administered the vaccine. Nearly half (49.2%) reported never discussing RSV vaccination, and two-thirds (66.7%) were unsure about vaccine availability at their workplaces (Table 2).

**Table 2:** Assessment of knowledge, attitudes, and practices toward RSV vaccine (n=120) Attitude items have a response category ranging from "strongly disagree" coded as 1 to "strongly agree" coded as 5. †Absolute question for the assessment of practice. RSV: respiratory syncytial virus

Knowledge item	N (%)
RSV is a seasonal virus similar to influenza. (True)	90 (75%)
RSV infection in older adults can lead to complications such as pneumonia and exacerbation of chronic illnesses. (True)	105 (87.5%)
The RSV vaccine is recommended for adults aged 50 years and older. (True)	83 (69.2%)
The RSV vaccine can reduce hospitalization due to lower respiratory tract infections in older adults. (True)	102 (85%)
The RSV vaccine is recommended by the Saudi Ministry of Health. (True)	77 (64.2%)
Only one RSV vaccine is currently approved for use in older adults in Saudi Arabia. (False)	17 (14.2%)
RSV vaccination can be co-administered with influenza and COVID-19 vaccines. (True)	69 (57.5%)
Total knowledge score (mean±SD)	4.68±1.91
Level of knowledge	
Poor	31 (25.8%)
Moderate	39 (32.5%)
Good	50 (41.7%)
Attitude: Please indicate your level of agreement with the following statements	Mean±SD
RSV is a serious disease in older adults.	4.14±0.91
I am confident in my knowledge about RSV vaccination in older adults.	3.28±1.18
RSV vaccination should be part of routine preventive care in older adults.	4.04±0.93
I feel confident discussing RSV vaccination with my patients.	3.28±1.26
I would recommend RSV vaccination to my older patients if it is available.	3.93±0.98
Total attitude score (mean±SD)	18.7±4.51
Level of attitude	
Negative	32 (26.7%)
Neutral	45 (37.5%)
Positive	43 (35.8%)
Practice items	N (%)
Have you ever discussed RSV vaccination with an older patient?^†^	
Yes	35 (29.2%)
No	85 (70.8%)
Have you ever prescribed or administered an RSV vaccine to an older patient?	
Yes	17 (14.2%)
No	103 (85.8%)
How often do you discuss RSV vaccination with eligible patients?	
Never	59 (49.2%)
Rarely	26 (21.7%)
Sometimes	21 (17.5%)
Often	12 (10%)
Always	2 (1.7%)
Are RSV vaccines currently available at your workplace?	
Yes	13 (10.8%)
No	27 (22.5%)
Not sure	80 (66.7%)

**Figure 1:**
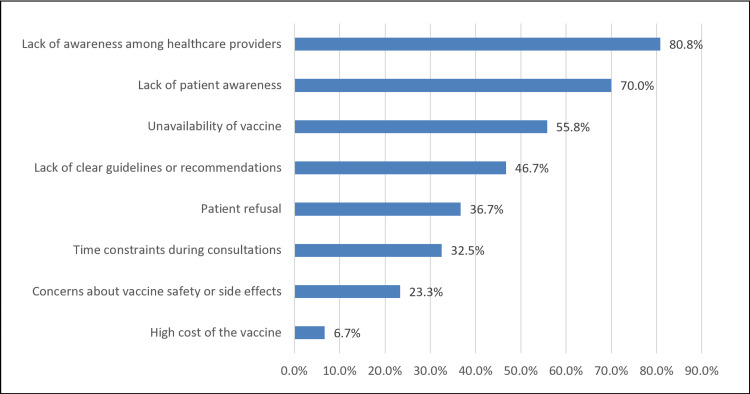
Main barriers preventing RSV vaccination in older adults RSV: respiratory syncytial virus

The main barriers perceived by practitioners were a lack of awareness among healthcare providers (80.8%) and patients (70%), followed by vaccine unavailability (55.8%) and unclear guidelines (46.7%). Patient refusal (36.7%) and safety concerns (23.3%) were also noted, whereas high costs were the least reported barriers (6.7%) (Figure 1).

**Figure 2:**
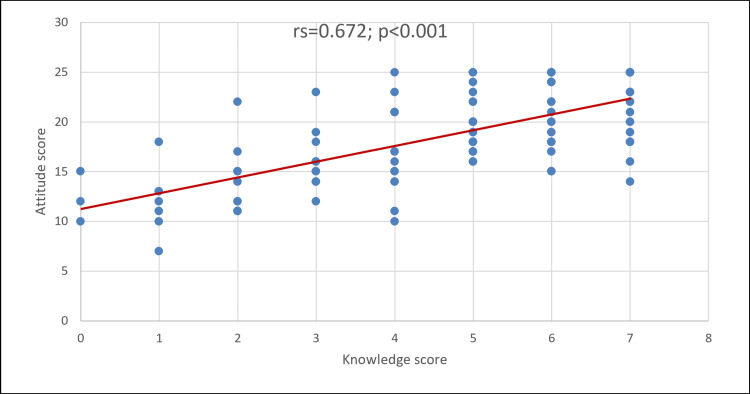
Correlation between knowledge and attitude scores

A strong positive correlation was observed between knowledge and attitude scores (Spearman's r=0.672; p<0.001). Practitioners with more knowledge tended to express more positive attitudes toward RSV vaccination (Figure 2).

The practice of RSV vaccination was significantly associated with years of experience, job position, knowledge, and attitude. Consultants/registrars were more likely to discuss RSV vaccination than residents/general practitioners (p < 0.001). Similarly, those with good knowledge (71.4%) and positive attitudes (77.1%) were significantly more likely to engage in favorable practices than those with poor knowledge or negative attitudes (p<0.001). No significant associations were found between age, gender, and job position (p>0.05) (Table 3).

**Table 3:** Relationship between the demographic characteristics, knowledge, attitudes, and practices regarding RSV vaccination in older adults (n=120) §P-value has been calculated using Fisher's test. **Significant at p<0.05 level. RSV: respiratory syncytial virus

Factor	Practice of RSV		P-value§
	Yes N (%) (n=35)	No N (%) (n=85)	
Age group			
≤35 years	17 (48.6%)	56 (65.9%)	0.100
>35 years	18 (51.4%)	29 (34.1%)	
Gender			
Male	22 (62.9%)	40 (47.1%)	0.159
Female	13 (37.1%)	45 (52.9%)	
Job position			
Resident/general practitioner	6 (17.1%)	45 (52.9%)	0.001
Consultant/registrar	29 (82.9%)	40 (47.1%)	
Years of experience in family medicine			
≤10 years	19 (54.3%)	64 (75.3%)	0.030**
>10 years	16 (45.7%)	21 (24.7%)	
Level of knowledge			
Poor	1 (2.9%)	30 (35.3%)	<0.001**
Moderate	9 (25.7%)	30 (35.3%)	
Good	25 (71.4%)	25 (29.4%)	
Level of attitude			
Negative	0 (0.0%)	32 (37.6%)	<0.001**
Neutral	8 (22.9%)	37 (43.5%)	
Positive	27 (77.1%)	16 (18.8%)	

Knowledge scores did not differ significantly by demographic characteristics; however, practitioners with more than 10 years of experience had higher mean scores (5.14 vs. 4.48), although this difference did not reach statistical significance (p=0.067) (Table 4).

**Table 4:** Differences in knowledge scores in relation to the demographic characteristics of the family medicine practitioners (n=120) §P-value has been calculated using the Mann-Whitney Z-test.

Factor	Knowledge score (7) mean±SD	Z-test	P-value^§^
Age group			
≤35 years	4.48±1.90	1.620	0.105
>35 years	5.00±1.91		
Gender			
Male	4.92±1.88	1.553	0.120
Female	4.43±1.94		
Job position			
Resident/general practitioner	4.61±2.06	1.139	0.255
Consultant/registrar	5.18±1.87		
Years of experience in family medicine			
≤10 years	4.48±1.94	1.831	0.067
>10 years	5.14±1.79		

Attitude scores differed significantly according to years of experience (p=0.037). Practitioners with more than 10 years of experience had more positive attitudes (mean=20.0) than those with less experience (mean=18.1). No significant differences were observed by age, gender, or job position (p>0.05) (Table 5).

**Table 5:** Differences in attitude scores in relation to the demographic characteristics of the family medicine practitioners (n=120) §P-value has been calculated using the Mann-Whitney Z-test. **Significant at p<0.05 level.

Factor	Attitude score (25) mean±SD	Z-test	P-value^§^
Age group			
≤35 years	18.1±4.43	1.538	0.124
>35 years	19.5±4.56		
Gender			
Male	19.2±4.90	1.410	0.158
Female	18.1±4.02		
Job position			
Resident/general practitioner	19.2±4.27	1.058	0.290
Consultant/registrar	20.2±4.51		
Years of experience in family medicine			
≤10 years	18.1±4.39	2.091	0.037**
>10 years	20.0±4.56		

## Discussion

Demographic context

This study examined family medicine practitioners' preparedness for RSV vaccination implementation among older adults, as reflected in their knowledge, attitudes, and reported practices. Most participants were at an early stage in their careers with less than five years of experience, which may have affected the results. Compared to various surveys of broader healthcare groups, participants were younger and less experienced. This may explain the moderate level of knowledge and limited practices observed in this study. Many participants had limited exposure to the updated guidelines and fewer opportunities to administer vaccines, which likely influenced their responses [11,12,18,20,21].

Knowledge levels

Knowledge levels varied substantially across the domains. While participants demonstrated awareness of RSV severity in older adults, gaps persisted regarding vaccine indications, availability, and co-administration guidelines [18]. They understood that RSV is seasonal and affects older adults, but many did not know the Ministry of Health recommendations or whether vaccines could be administered together. Consistent with our reports, studies from the Middle East have also found limited awareness of RSV and its vaccines, with many unaware of the FDA-approved options [9,22]. This, however, differs from US and European studies, in which healthcare professionals were more familiar with RSV in older adults, although gaps in vaccine recommendations persisted [1,3,4,6,7,10]. These differences may reflect variation in vaccine rollout strategies, guideline dissemination, and the professional composition of surveyed samples [11,21,23].

Attitudes toward vaccination

Attitudes toward RSV vaccination reflect cautious optimism. Although clinicians acknowledge RSV as a significant clinical threat, their limited confidence in counseling patients suggests gaps in perceived preparedness for implementation. Other studies have shown that healthcare professionals generally support RSV prevention and vaccination, although concerns about its safety and side effects linger [8,12,24]. Lower confidence in this group may be due to limited clinical experience and uncertainty regarding vaccine availability. Studies with more experienced participants had better vaccine advocacy and provided stronger support for vaccination [11,20,21]. These results suggest that clinical experience and institutional support help build confidence and the willingness to recommend vaccines.

Practice behaviors

Despite moderate knowledge of RSV, its translation into clinical practice remains limited. Few practitioners have proactively discussed vaccination with older adults, highlighting a disconnect between knowledge and practice, which mirrors the findings of international studies. In this study, only 14.2% of respondents had prescribed or administered RSV vaccines, and almost half said they had never discussed vaccination with patients. US and European studies have shown greater involvement, with doctors recommending RSV vaccinations to older adults more frequently [5,12,20]. Systemic barriers may explain this difference, as many participants were unsure of vaccine availability and found the guidelines unclear [18]. These barriers seem stronger in this setting than in other countries, where better access and clearer recommendations may have led to higher vaccination rates [14,15,18]. Variations in the way vaccines are introduced, along with the degree of available institutional support, likely influenced these discrepancies.

Perceived barriers

The most frequently reported barriers reflected system-level constraints, particularly lack of provider awareness, inconsistent vaccine availability, and unclear institutional guidelines, factors that impede vaccine uptake irrespective of the physician's intent. Other studies have identified safety concerns, patient hesitancy, and limited awareness as important barriers [14,15,13]. In contrast, cost was rarely mentioned in this study, whereas international research has often highlighted affordability and reimbursement as major issues [14,15]. This variation may be explained by differences in healthcare funding and vaccine policies in different regions; therefore, cost is less of a problem in this setting [9,16,17].

Explaining differences with published literature

Several factors explain why these findings differ from those in other published reports. This study mostly included younger and less-experienced practitioners, whereas other studies included more senior doctors and pharmacists [11,12,21,23]. Differences in how vaccines are introduced, how guidelines are shared, and how healthcare systems work also affect awareness and practice [9,10,22]. Timing also matters, since this study took place early in the RSV vaccine rollout, while other surveys were conducted after recommendations were more widely shared [6,7,18,20]. Systemic barriers, such as vaccine unavailability and unclear guidelines, were greater issues here, while cost and safety concerns were more critical elsewhere [14,15,13]. These differences highlight the need to adapt interventions to local needs while learning from the global experience.

Study limitations

This study had several limitations. First, the sample included only family medicine practitioners from one institution and region; therefore, the results may not apply to other healthcare settings or specialties. Most participants were early-career doctors with less than five years of experience, which may have led to lower knowledge and practice levels than those of more experienced physicians. Second, because the study was cross-sectional, it captured attitudes and practices at a single point in time and did not show changes as RSV vaccines became more widely available and guidelines were updated. Third, using self-reported practices may have introduced recall or social desirability bias, potentially affecting the accuracy of the reported behaviors. Finally, this study did not examine patient outcomes, such as vaccine uptake or satisfaction, which would help demonstrate the impact of physicians' knowledge and attitudes.

Future direction

Future research can expand on these findings by examining how RSV vaccination practices change as national guidelines are updated and vaccines become more widely available. Long-term studies are important for tracking changes in physicians' knowledge, confidence, and clinical behavior, especially as new doctors enter the field and policies become clearer. Conducting studies across different centers and healthcare settings in Saudi Arabia could determine whether the barriers identified in this study are common nationwide or specific to certain settings. Adding qualitative research, such as in-depth interviews or focus groups, could provide deeper insight into the daily challenges doctors face during consultations, including limited time, competing priorities, and the difficulty of communicating effectively with older adults. Future studies would benefit from examining patient‑level outcomes, such as actual vaccine uptake, satisfaction with the vaccination process, and the degree of trust patients place in preventive care, to better understand how physicians' preparedness translates into real protection for older adults. Examining these elements can guide the development of more tailored strategies and strengthen the evidence base for improving RSV prevention efforts in primary care settings.

## Conclusions

This study highlights the substantial gaps in knowledge, confidence, and clinical practices regarding RSV vaccination among family medicine practitioners. Systemic barriers, such as vaccine unavailability and unclear guidelines, played a major role. The strong correlation between knowledge and attitude underscores the importance of clinician preparedness and highlights the need for targeted education to support physicians' confidence in and involvement in vaccine advocacy; however, education alone is unlikely to address implementation gaps without accompanying system-level supports. The differences between these findings and those of other studies are likely due to population characteristics, regional contexts, and the timing of vaccine introduction, underscoring the need for context-specific strategies. Improving provider and patient awareness, clarifying guidelines, and ensuring vaccine availability, along with clinician support embedded within primary care workflows, are the key steps in closing the gap between knowledge and practice. Building the confidence and readiness of physicians, supported by these coordinated measures, is necessary to increase RSV vaccine uptake and reduce the impact of RSV in older adults and high-risk groups.

## Appendices

**Table 6:** Study questionnaire RSV: respiratory syncytial virus

Section	Q# (within section)	Question/Item	Response options
Consent and Introduction	-	Consent/intro text (study title, purpose, time, voluntariness, anonymity/confidentiality, PI contact)	"Yes" to begin
Section 1: Demographics	1	Age (years)	<30; 30-35; 36-40; 41-45; 46-50; >50
	2	Gender	Male; Female
	3	Current position	Consultant; Registrar; Resident; General practitioner
	4	Years of clinical practice	<5; 5-10; 11-15; 16-20; >20
Section 2: Knowledge and Awareness (True/False/I don't know)	1	RSV is a seasonal virus similar to influenza	True; False; I don't know
	2	RSV infection in older adults can lead to complications such as pneumonia and exacerbation of chronic illnesses	True; False; I don't know
	3	The RSV vaccine is recommended for adults aged 50 years and older	True; False; I don't know
	4	The RSV vaccine can reduce hospitalization due to lower respiratory tract infections in older adults	True; False; I don't know
	5	The RSV vaccine is recommended by the Saudi Ministry of Health	True; False; I don't know
	6	Only one RSV vaccine is currently approved for use in older adults in Saudi Arabia	True; False; I don't know
	7	RSV vaccination can be co-administered with influenza and COVID-19 vaccines	True; False; I don't know
Section 3: Attitudes (1=strongly disagree → 5=strongly agree)	1	RSV infection is a serious disease in older adults	1-5 Likert scale
	2	I am confident in my knowledge about RSV vaccination in adults	1-5 Likert scale
	3	RSV vaccination should be part of routine preventive care in older adults	1-5 Likert scale
	4	I feel confident discussing RSV vaccination with my patients	1-5 Likert scale
	5	I would recommend RSV vaccination to my older patients if it is available	1-5 Likert scale
Section 4: Practices and Barriers	1	Have you ever discussed the RSV vaccination with an older adult?	Yes; No
	2	Have you ever prescribed or administered an RSV vaccine to an older adult?	Yes; No
	3	How often do you discuss RSV vaccination with eligible patients?	Always; Often; Sometimes; Rarely; Never
	4	Are RSV vaccines currently available at your workplace?	Yes; No; Not sure
	5	What are the main barriers to RSV vaccine administration in your practice? (Select all that apply)	Lack of awareness among healthcare providers; Lack of patients' awareness; Unavailability of vaccine; High cost of the vaccine; Lack of clear guidelines or recommendations; Time constraints during consultations; Concerns about vaccine safety or side effects; Patient refusal; Other: specify
